# Collateral rostral thalamic projections to prelimbic, infralimbic, anterior cingulate and retrosplenial cortices in the rat brain

**DOI:** 10.1111/ejn.15819

**Published:** 2022-09-23

**Authors:** Steliana Yanakieva, Mathias L. Mathiasen, Eman Amin, Andrew J. D. Nelson, Shane M. O'Mara, John P. Aggleton

**Affiliations:** ^1^ School of Psychology Cardiff University Wales UK; ^2^ Department of Veterinary and Animal Sciences University of Copenhagen Frederiksberg Denmark; ^3^ Institute of Neuroscience Trinity College Dublin Ireland

**Keywords:** anatomy, anterior thalamus, cingulate cortex, frontal cortex, nucleus reuniens, prelimbic cortex, thalamus

## Abstract

As the functional properties of a cortical area partly reflect its thalamic inputs, the present study compared collateral projections arising from various rostral thalamic nuclei that terminate across prefrontal (including anterior cingulate) and retrosplenial areas in the rat brain. Two retrograde tracers, fast blue and cholera toxin B, were injected in pairs to different combinations of cortical areas. The research focused on the individual anterior thalamic nuclei, including the interanteromedial nucleus, nucleus reuniens and the laterodorsal nucleus. Of the principal anterior thalamic nuclei, only the anteromedial nucleus contained neurons reaching both the anterior cingulate cortex and adjacent cortical areas (prefrontal or retrosplenial), though the numbers were modest. For these same cortical pairings (medial prefrontal/anterior cingulate and anterior cingulate/retrosplenial), the interanteromedial nucleus and nucleus reuniens contained slightly higher proportions of bifurcating neurons (up to 11% of labelled cells). A contrasting picture was seen for collaterals reaching different areas within retrosplenial cortex. Here, the anterodorsal nucleus, typically provided the greatest proportion of bifurcating neurons (up to 15% of labelled cells). While individual neurons that terminate in different retrosplenial areas were also found in the other thalamic nuclei, they were infrequent. Consequently, these thalamo‐cortical projections predominantly arise from separate populations of neurons with discrete cortical termination zones, consistent with the transmission of segregated information and influence. Overall, two contrasting medial‐lateral patterns of collateral projections emerged, with more midline nuclei, for example, nucleus reuniens and the interoanteromedial nucleus innervating prefrontal areas, while more dorsal and lateral anterior thalamic collaterals innervated retrosplenial cortex.

## Graphical abstract

The study identified collateral projections arising from rostral thalamic nuclei that terminate across prefrontal (including anterior cingulate) and retrosplenial areas in the rat brain. The analysis focused on the individual anterior thalamic nuclei, the interanteromedial nucleus, nucleus reuniens and the laterodorsal nucleus. Neurons innervating both medial prefrontal/anterior cingulate cortices and anterior cingulate/retrosplenial cortices were most evident at or close to the thalamic midline, contrary to the neurons that collaterise to reach different parts of the retrosplenial cortex.

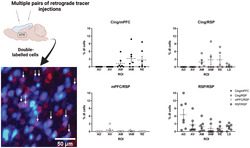

**Abbreviations**
AD: anterodorsal nucleus
AM: anteromedial nucleus
AV: anteroventral nucleus
cb: cingulum bundle
Cg1: cingulate cortex area 1
Cg2: cingulate cortex area 2
Cing/RSP: cingulate/retrosplenial group pairs
CTB: cholera toxin B
DLO: dorsolateral orbital cortex
DP: dorsal peduncular cortex
DS: dorsal subiculum
FB: fast blue
Fr3: fasciculus retroflexus 3
IAM: interanteromedial nucleus
IL: infralimbic cortex
LD: laterodorsal nucleus
LO: lateral orbital cortex
M1: primary motor cortex
M2: secondary motor cortex
MO: medial orbital cortex
MO: medial orbital cortex
mPFC/Cing: medial prefrontal/anterior cingulate group pairs
mPFC/RSP: medial prefrontal/retrosplenial group pairs
NA: not analysed
PBS: phosphate‐buffered saline
PFA: paraformaldehyde
Post: postsubiculum
PrL: prelimbic cortex
PT: parataenial nucleus
RE: nucleus reuniens
RSD: dysgranular retrosplenial cortex
RSG: granular retrosplenial cortex (a,b,c)
RSGa: granular retrosplenial cortex a
RSGb: granular retrosplenial cortex b
RSGc: granular retrosplenial cortex c
RSP: retrosplenial cortex (granular and dysgranular)
RSP/RSP: retrosplenial/retrosplenial group pairs
SUB: submedius thalamic nucleus
TT: tenia tecta
V1B: primary visual cortex, binocular area
V1M: primary visual cortex, monocular area
V2: secondary visual cortex.
VA: ventral anterior thalamic

## 1 INTRODUCTION

The function of any given cortical area is, in part, determined by its thalamic inputs (Jones, 2002; Sherman, 2007). Numerous tracer studies have identified those rat thalamic nuclei that project to frontal and cingulate regions such as the orbital, prelimbic, anterior cingulate and retrosplenial areas. These studies reveal that a range of nuclei, which include the anterior thalamic nuclei, the laterodorsal nucleus and nucleus reuniens project to more than one of these cortical areas (Condé et al., 1990; Hoover & Vertes, 2007; Krettek & Price, 1977; Shibata, 1993; Vertes et al., 2006; Wyss & Van Groen, 1992). Far less is known, however, about whether these diverse cortical inputs arise from segregated populations of thalamic neurons or whether they reflect collateral projections that enable individual neurons to influence different cortical areas simultaneously.

An initial investigation placed different retrograde tracers into the anterior cingulate and retrosplenial cortices (Horikawa et al., 1988). Between 8% and 14% of the labelled efferents in the three principal anterior thalamic nuclei (anterodorsal, anteromedial and anteroventral) projected to both the caudal anterior cingulate cortex and nearby rostral retrosplenial cortex (Horikawa et al., 1988). Lower percentages were seen when the cortical injection sites were increasingly separated in distance. Meanwhile, no laterodorsal nucleus neurons were observed projecting to both the anterior cingulate and retrosplenial cortices (Horikawa et al., 1988). No other thalamic nuclei were included in that study. A related investigation (Condé et al., 1990), which looked for inputs to the anterior cingulate cortex and other medial prefrontal areas, described how midline thalamic sites, such as nucleus reuniens contain modest numbers of bifurcating neurons. A more recent study (Pei et al., 2021) extended the termination areas under investigation by including the hippocampal formation but just focussed on projections from the anteromedial nucleus and nucleus reuniens. That study reported appreciably higher proportions of double‐labelled neurons that collaterise to terminate in multiple areas. For example, the proportion of anteromedial nucleus and nucleus reuniens labelled projections that innervated both the medial prefrontal cortex and dorsal subiculum was over 30% for both nuclei (Pei et al., 2021). Meanwhile over 19% of anteromedial nucleus and nucleus reuniens labelled efferents reached both the medial prefrontal cortex and caudal retrosplenial cortex (Pei et al., 2021). Related studies have observed bifurcating neurons from nucleus reuniens reaching both prefrontal cortex and the hippocampus, comprising <10% of labelled cells (Hoover & Vertes, 2012; Varela et al., 2014). Together these studies confirm that such collaterals exist but leave unreported various medial cortical terminal combinations alongside gaps in the rostral thalamic nuclei under investigation.

A closely related question is whether individual rostral thalamic neurons terminate in separate parts of the *same* cortical area. To address this question, pairs of retrograde tracers were injected, one within the rostral retrosplenial cortex, the other within the caudal retrosplenial cortex (Sripanidkulchai & Wyss, 1986). No double‐labelled cells were observed in the anterior thalamic nuclei or the laterodorsal nucleus despite considerable numbers of single‐labelled cells (Sripanidkulchai & Wyss, 1986). However, a very different outcome was described in a later study that also placed pairs of retrograde tracers in separate rostral and caudal retrosplenial locations (Horikawa et al., 1988). That study reported double‐labelled cells in all three anterior thalamic nuclei (anterodorsal, anteromedial and anteroventral), although extremely few were observed in the laterodorsal nucleus (Horikawa et al., 1988). These double‐labelled cells were most numerous in the anterodorsal nucleus (up to 22% of labelled cells). These two conflicting sets of results (Horikawa et al., 1988; Sripanidkulchai & Wyss, 1986) leave uncertain whether individual anterior thalamic projections collaterise across retrosplenial cortex. This issue should be resolved given the significance of these thalamo‐cortical projections for learning and memory (Vann et al., 2009; Yamawaki et al., 2019).

In view of these uncertainties and the many gaps in our current knowledge, the present study further examined whether rostral thalamic neurons collaterise to reach separate medial cortical areas. Most authorities place the infralimbic, prelimbic, anterior cingulate and medial agranular cortices within the ‘medial prefrontal cortex’ (Hoover & Vertes, 2007; Öngür & Price, 2000; but see Conde et al., 1990). Given the need to separate anterior cingulate cortex tracer injections from those in other medial prefrontal areas, we have used the group designation ‘medial prefrontal’ for medial cortical areas ventral to the cingulate area. Consequently, different retrograde tracers were placed in four pairs of cortical areas: medial prefrontal/anterior cingulate (mPFC/Cing), anterior cingulate/retrosplenial (Cing/RSP), medial prefrontal/retrosplenial (mPFC/RSP) and retrosplenial/retrosplenial (RSP/RSP). We then counted single and double retrogradely labelled cell populations within the three principal anterior thalamic nuclei, the interanteromedial nucleus, nucleus reuniens and the laterodorsal nucleus, thereby adding to those nuclei included in previous investigations (Condé et al., 1990; Horikawa et al., 1988; Pei et al., 2021). These same thalamic nuclei are of related interest given their various roles in spatial learning and cognition (Aggleton et al., 2010; Cassel et al., 2021; Griffin, 2021; Mathiasen et al., 2021; Van Der Werf et al., 2002; van Groen et al., 2002).

## 2 MATERIALS AND METHODS

### 2.1 Animals

Thirty‐three Lister Hooded male rats (Envigo, UK) underwent surgery. Nine animals were excluded due to lack of spread of the tracer or injections beyond the target area. The data reported are from the remaining 24 animals. The rats were allocated in the four experimental groups as follows: (1) mPFC/Cing, *n* = 7; (2) Cing/RSP, *n* = 5; (3) mPFC/RSP, *n* = 5; (4) RSP/RSP, *n* = 7. At the time of the surgery the animals weighed between 284 and 663 g (*M* = 348.8 g). Prior to surgery, all animals were housed in pairs, in a temperature‐controlled room, under 12‐h light/dark cycle. Food and water were available ad libitum. All animals were randomly assigned to each group and underwent the same surgical procedures. All animal procedures were carried out in accordance with UK Animals (Scientific Procedures) Act 1986 and were approved by the local Ethics Committee at Cardiff University. Six of the cases were also included in an analysis of the topography of anteroventral nucleus projections to retrosplenial cortex (Lomi et al., 2021).

### 2.2 Surgeries and tracer infusions

All surgeries took place under isoflurane‐oxygen mixture anaesthesia (5% induction, 1.5–2.5% maintenance). Each rat was placed in a stereotaxic frame (David Kopf Instruments, CA, USA), so that the skull was flat. Chloramphenicol .5% eye‐gel was applied, meloxicam (.06 ml) was administered subcutaneously for analgesic purposes, and lidocaine (.1 ml of 20 mg/ml solution) was applied topically to the incision site. Craniotomies were made over the right hemisphere in 21 animals and in three animals (from the Cing/RSP group) over the left hemisphere. The anterior cingulate injections deliberately targeted the more rostral parts of this area to avoid the transitional, midcingulate area 24′ (Vogt & Paxinos, 2012).

Combinations of two retrograde tracer injections at different cortical locations were used. The tracers were fast blue (FB, Sigma‐Aldrich, Gillingham, UK; 3% solution in PBS) and non‐conjugated cholera‐toxin b (CTB, List Biological Laboratories Inc, CA; 1% solution in .05‐M tris). These two tracers exhibit different patterns when filling neuronal cell bodies (Köbbert et al., 2000). All FB injections were made mechanically and CTB injections were made either mechanically (22 cases) or iontophoretically (2 cases). All mechanical injections used a 1.0‐μl Hamilton Syringe (Hamilton, Bonaduz, Switzerland). A dedicated syringe was used for FB and another for CTB. The tracers were infused at a flow rate of 20 ηl/min for 3–5 min, and the needle was left in situ for further 5 min before retraction. Iontophoretic injections were infused by a 6 s on/off pulse with 2‐, 6‐ and 7‐μA current for approximately 5 min each setting (total 15 min). The volume of the FB injections was 25–150 ηl (*M* = 73.75 ηl) and for CTB injections was 60–180 ηl (*M* = 78.33 ηl).

At the end of the surgeries, analgesic lidocaine and antibiotic powder (Clindamycin, Pfizer, UK) were applied to the surgical site. All animals were subcutaneously administered 5‐ml glucose‐saline solution for fluid replacement, prior to placing them in a recovery chamber. When conscious, the animals were returned to their home cage and closely monitored until they were sacrificed.

### 2.3 Perfusions

After a survival time of 6–8 days, the animals received a lethal injection of sodium phenobarbital (2 ml/kg, Euthatal, Marial Animal Health, UK) administered intraperitoneally. Then, the animals were transcardially perfused with .1‐M phosphate‐buffered saline (PBS) and 4% paraformaldehyde in .1 M PBS (PFA). The brains were further post‐fixed in PFA for at least 2 h, and then placed in 25% sucrose solution for minimum of 24 h. The tissue was cut into 50‐μm coronal sections in 4 series (i.e., 1 in 4), using a freezing microtome (8000 Sledge Microtome, Bright Instruments). The tissue was stored in cryoprotectant (30% sucrose, 1% polyvinyl pyrrolidone, 30% ethylene glycol in PBS) in a freezer at −20°C.

### 2.4 Histology

For CTB staining, the sections were washed for 3 × 10 min in a .1‐M PBS, followed by 3 × 10 min washes in PBST (.2% Triton X‐100 in .1‐M PBS). Sections were then incubated with the primary antibody rabbit‐anti‐CTB overnight (1:3000) (Sigma‐Aldrich, UK) for 16–24 h at room temperature. The sections were then washed three times for 10 min with PBST and transferred to a secondary antibody of goat‐anti‐rabbit (Dylight Alexa flour 594, Vector Laboratories, Peterborough, UK) for 2 h on a stirrer. Finally, the sections were washed with PBS and mounted onto gelatin‐coated slides and cover‐slipped using Fluromount (Sigma‐Aldrich, Germany) or DPX (Thermo Fisher, Waltham, MA) mounting medium.

Where necessary to confirm the boundaries of the regions of interest, an additional series was mounted onto gelatin‐coated slides and Nissl‐stained using cresyl violet. The sections were then dehydrated through increasing concentrations of alcohol (70%; 90%; 100%; 100%) and washed in xylene. Then, the slides were cover‐slipped with DPX (Thermo Fisher, Waltham, MA) mounting medium.

### 2.5 Image acquisition

Sections were viewed in the dark using a Leica DM5000B fluorescent microscope. Images of the regions of interest and the injection sites were acquired using Leica DFC310FX digital camera in the Leica Application Suite. An A4 DAPI filter was used to view the FB label and N21 filter for the CTB label. The images were acquired at magnifications of 5× and/or 10× for single channels and a combined overlay of both channels. Photomicrographs acquired for illustration purposes were occasionally adjusted for contrast, brightness, and intensity.

### 2.6 Cell counts

While stereological methods are essential to derive absolute cell counts (Coggeshall & Lekan, 1996), the present study sought to compare the relative numbers of double‐labelled cell profiles within the regions of interests, between the four group. For this purpose, non‐stereological methods are appropriate when certain conditions are met (e.g., random tissue sampling is used and there are no systematic changes in the volume or packaging of neurons across different regions) (Coggeshall & Lekan, 1996). In the present study we targeted the entire thalamic nucleus, counteracting the need for random tissue sampling within a given region of interest. Although, the volume and packaging of cells undoubtedly vary between different nuclei, the focus here centres on the proportions of double‐labelled profiles within and between the regions of interest, rather than the absolute number of profiles. This same focus meant that we did not attempt to estimate total neuronal numbers within a given nuclei, for example, after Nissl staining.

With these constraints in mind, profiles of cells were counted manually using Image J 1.53 (Rasband, 2011, NIH, USA). Single‐labelled FB and CTB cells were counted across each region of interest within the three major anterior thalamic nuclei (the anterodorsal, anteroventral and anteromedial thalamic nuclei) as well as the interanteromedial nucleus and nucleus reuniens. Additionally, cell counts were made in the laterodorsal nucleus for the Cing/RSP and RSP/RSP pairs. Laterodorsal cell counts were not made for the mPFC injection combinations as that nucleus does not project to the infralimbic, prelimbic, or rostral anterior cingulate cortices (Condé et al., 1990; Hoover & Vertes, 2007).

The fluorescent labelled cells within the entire regions of interest were counted separately for each tracer. The boundaries of the regions were guided by the Paxinos and Watson rat atlas (5th edition, 2004). A minimum of two sections per region were counted for each case (*M* = 3). ‘Double‐labelled neurons’ were defined as those showing evident blue FB label at the centre of the cell body surrounded by red (CTB) ring‐like label. Where it was unclear whether a neuron was double‐labelled, Corel Draw 2019 (Corel Corporation, USA) was used to create an overlay between the two separate channels and the opacity option was used to gradually transition between the single channel images, confirming the identical location and shape of the double‐labelled neurons. Next, double‐labelled cells were also counted in a subset of cases (at least one case per group) by a second researcher, blind to the original counts, to confirm inter‐rater reliability (*r* = .935, *p* < .01). The experimenters were not blinded to the group membership of the animals or the purpose of the experiment.

To further validate the double‐labelled counts, we used a Zeiss LSM880 Airyscan confocal microscope to acquire higher magnification images (20× and 40× magnification) from a minimum of two sections of each of the principal thalamic nuclei. Data were obtained from a subset of cases (225#8, 232#16, 604#5) that represented different cortical combinations, each with relatively high proportions of double‐labelled cells (confocal images were not acquired for a mPFC/RSP pair as double‐labelling was almost absent). Using a maximum intensity image projection, the same counting procedure and proportion estimation methods were used as described above. Where it was unclear if a cell was double‐labelled, a z‐stack of images of the corresponding region was inspected.

### 2.7 Analysis of double‐labelled cell counts

To provide a single measure for each thalamic nucleus we first calculated the total number of labelled cells for each case (cell numbers from both individual tracers added to the number of double‐labelled cells for that same region). This total was then used as a denominator to determine the percentage of double‐labelled cells with respect to all labelled cells (i.e., within a region of interest, the number of double‐labelled cells, was divided by the sum of just FB, just CTB and all double‐labelled cells). This measure of collaterisation is the same as that used in previous studies (Condé et al., 1990; Horikawa et al., 1988). To avoid violations to the assumptions of parametric tests, a series of non‐parametric Friedman tests helped to compare the proportions of double‐labelled cells between nuclei, within each injection pairing.

For a more complete picture we also calculated the percentage of double‐labelled cells in each thalamic nucleus with respect to the number of single‐labelled cells from each of the two injections in that same case, that is, the two separate counts of single‐labelled cells in each case (Table 1). Because there is only one number of double‐labelled cells in an individual animal, the proportion of double‐labelled cells will always be highest for the tracer injection resulting in the smaller number of single‐labelled cells (Table 1).

**TABLE 1:** Mean percentages of double‐labelled cells in each thalamic nucleus relative to the numbers of single‐labelled cells (either FB or CTB) resulting from each separate cortical injection

	mPFC/Cing		Cing/RSP		mPFC/RSP		RSP/RSP	
	% of mPFC cells	% of Cing cells	% of Cing cells	% of RSP cells	% of mPFC cells	% of RSP cells	% of rostral RSP cells	% of caudal RSP cells
**AD**	0	0	0	0	0	0	17 (1–46)	13.3 (1–27)
**AV**	0	0	0	0	1.4 (0–7)	1 (0–5)	6.7 (.7–23)	10.4 (1–38)
**AM**	3.3 (0–8)	3.1 (0–17)	7.9 (0–19)	6 (0–20)	.1 (0–.1)	.5 (0–2)	5.7 (0–17)	9.2 (0–46)
**IAM**	12.2 (0–47)	5.8 (0–24)	3.3 (0–11)	12.4 (0–45)	.12 (0–1)	4 (0–20)	2.2 (0–12)	6.6 (0–39)
**RE**	5.5 (0–24)	4.9 (0–14)	7 (0–15)	4.9 (0–14)	0	0	1.7 (0–6)	5.4 (0–21)
**LD**	NA	NA	1.4 (0–4)	.7 (0–2)	NA	NA	6.1 (0–13)	2.45 (0–5)

*Note*: From left to right, the proportions of double‐labelled cells with respect to the single‐labelled cell counts for each injection site within the medial prefrontal/anterior cingulate group pairs (mPFC/Cing), anterior cingulate/retrosplenial group pairs (Cing/RSP), medial prefrontal/retrosplenial group pairs (mPFC/RSP) and retrosplenial/retrosplenial group pairs (RSP/RSP) pairings. Since the number of double‐labelled cells is the same for both injection sites, the percentage of double‐labelled cells will always be highest with respect to the cortical area associated with the smaller numbers of single‐labelled cells. The numbers shown for each thalamic nucleus are the mean percentages of double‐labelled cells for all cases within that pairing. The numbers in brackets are the minimum and maximum percentages of double‐labelled cells observed within that group of cases (to nearest whole integer). See list of abbreviations.

## 3 RESULTS

Although cell counts are provided for all cases, the focus is on those cases giving the highest numbers and proportions of double‐labelled cells for a given combination of cortical injections. This focus reflects how the technique will inevitably underestimate the full extent of bifurcating neurons as the tracer injections are discrete; that is, they do not completely fill a given cortical area. Care was also taken to ensure that the injection sites in an individual case did not overlap. A further concern was the possibility of direct tracer uptake by the cingulum bundle following cingulate and retrosplenial injections. For this reason, we compared the observed distribution of retrograde label with past, published topographies (Perry et al., 2021; Shibata, 1993; Sripanidkulchai & Wyss, 1986). In no case did we observed patterns of retrograde label indicative of direct cingulum uptake (Bubb et al., 2020).

### 3.1 Collateral projections to both medial prefrontal and anterior cingulate cortices (mPFC/Cing)

**FIGURE 1:**
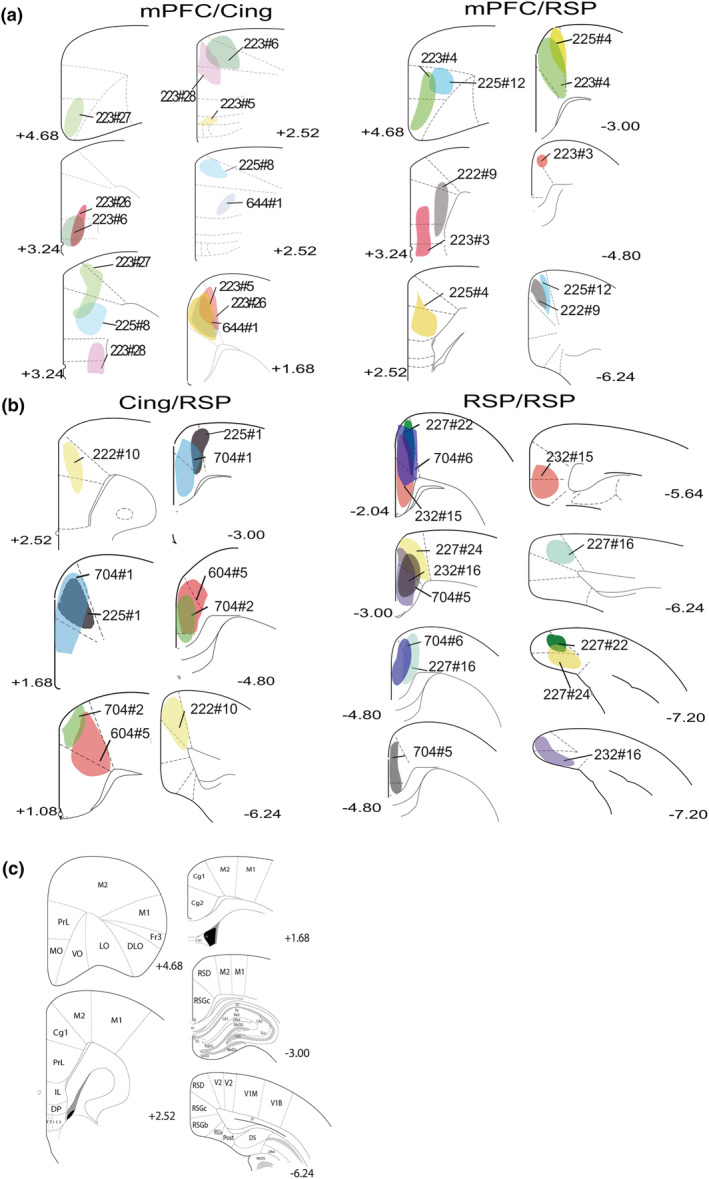
Schematic representation of the core of each injection site for the individual animals in each of the four groupings. Panel (a) shows those cases with a medial prefrontal cortex (mPFc) injection combined with either anterior cingulate (Cing) or retrosplenial cortex (RSP) injections. Panel (b) shows the injection sites for the Cing/RSP pairings, as well as the RSR/RSP pairings. Panel (c) shows annotated coronal sections that help to locate the various injection sites (sections based on Paxinos & Watson, 2004). A given animal has the same colour and case number within each of the four injection pairings. Other numbers refer to the location of the centre of the injections on the anterior–posterior axis (from bregma in mm). See list of abbreviations.

**FIGURE 2:**
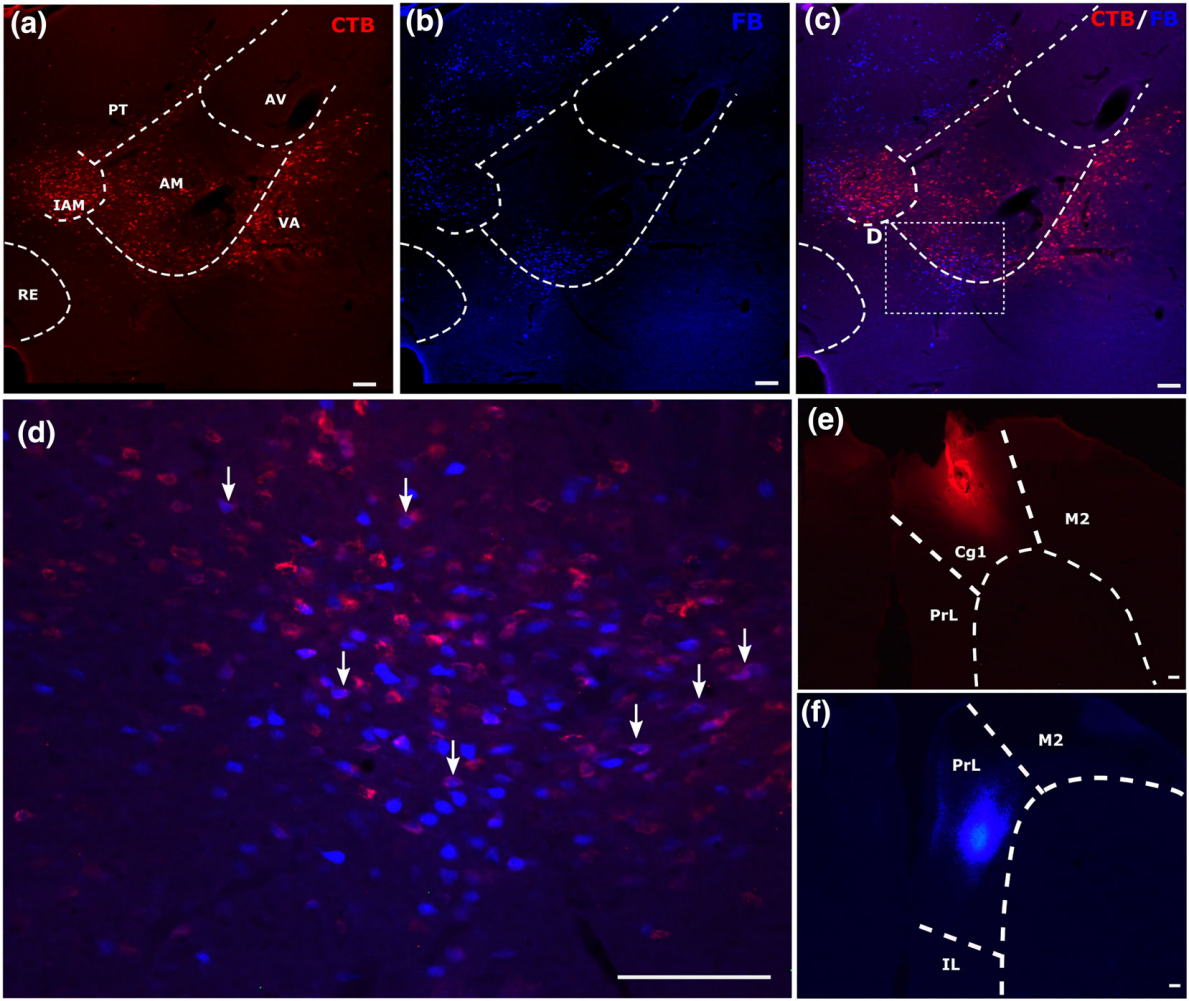
Example of combined medial prefrontal (FB) and anterior cingulate (CTB) tracer injections (case 225#8). Coronal sections containing the various thalamic nuclei under investigation. The upper panels (a–c) show label from the CTB (a) and FB (b) injections, as well as the overlay of the two channels (c). Panels (e) and (f) depict the CTB and FB injection sites, respectively. The enclosed area in panel C shows that part of the section magnified in panel (d). The arrows in panel (d) point to examples of double‐labelled cells, which show a blue centre (FB label) surrounded by red halo (CTB label). All scale bars are 150 μm. See list of abbreviations.

**FIGURE 3:**
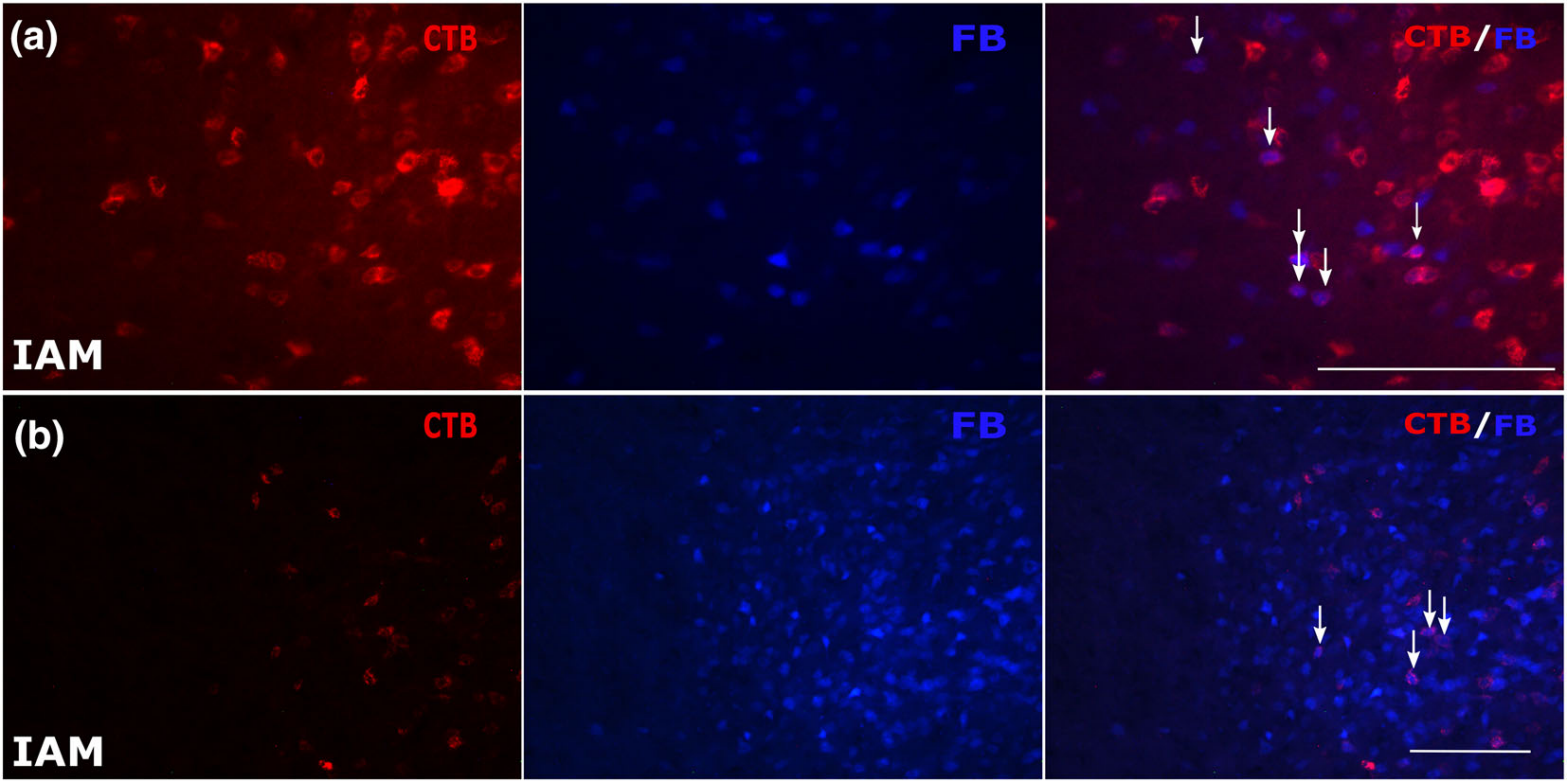
Labelling in the interanteromedial nucleus. Panels in row (a) show a portion of the interoanteromedial nucleus (case 225#8) in a mPFC/Cing pair. Panels in row (b) show a portion of the interanteromedial nucleus (case 604#5) in a Cing/RSP pair. Both panels show individual CTB and FB channels, with the overlays of the two pointing to double‐labelled cells. All scale bars are 150 μm. See list of abbreviations.

In seven cases (Figures 1, 2, 3) a retrograde tracer was infused into different portions within the medial prefrontal cortex, combined with a second retrograde tracer into the anterior cingulate cortex. The medial prefrontal cortex injections variously included the ventral prelimbic cortex, dorsal prelimbic cortex, infralimbic cortex, dorsal peduncular cortex, medial orbital cortex and tenia tecta. In one case (223#27) the more dorsal injection involved both the anterior cingulate and prelimbic cortices. Consequently, the two tracers included adjacent portions of prelimbic cortex, but at different heights. Given the potential for tracer overlap it is notable that the double‐labelled cell counts in this case were representative of the group.

**FIGURE 4:**
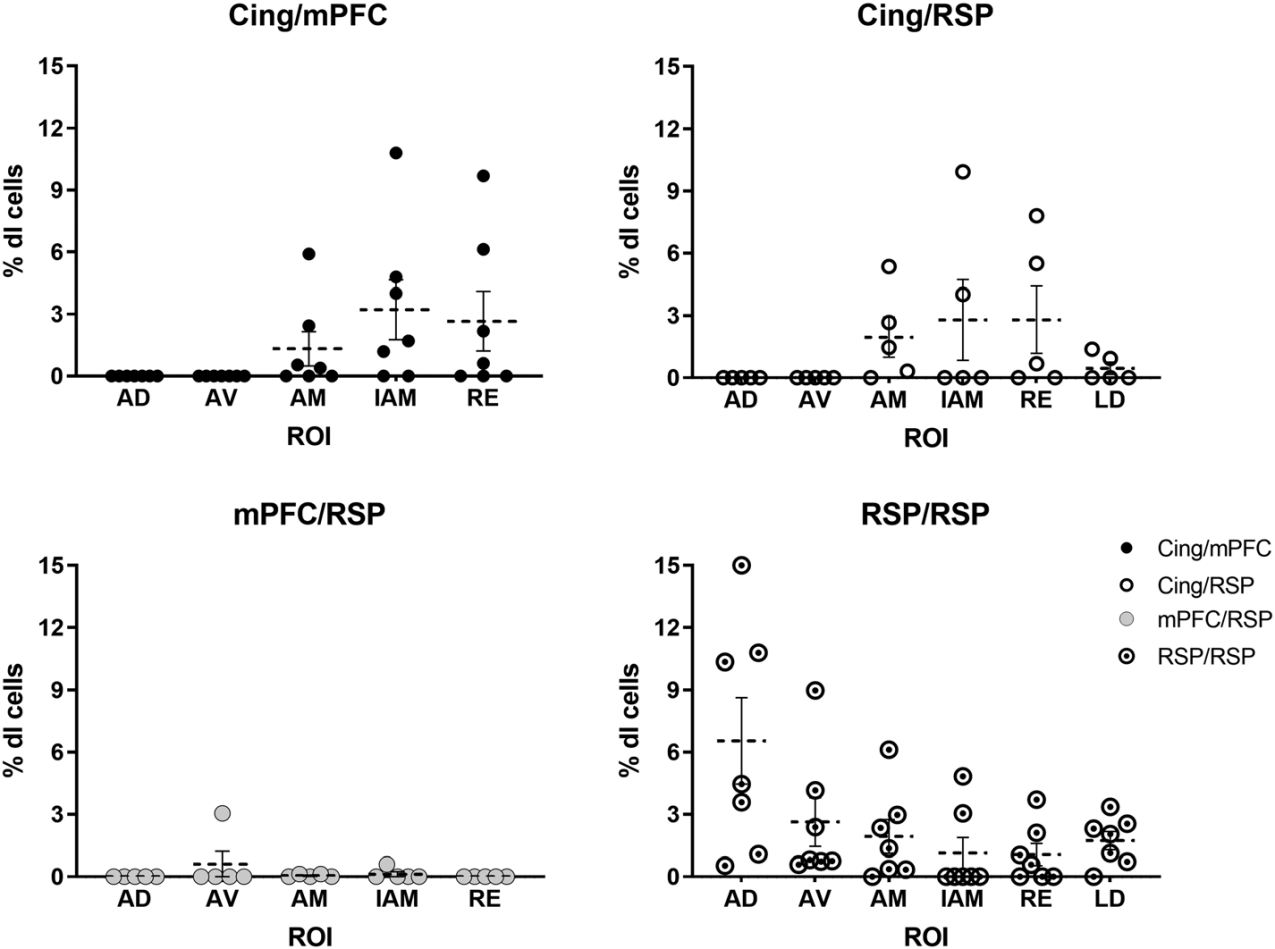
Proportion of double‐labelled cells relative to all single‐labelled cells (FB and CTB cell counts combined), expressed as percentages in the regions of interest (ROI) for each rat. The different symbols (black circles, white circles, grey circles, white circles with dot) indicate individual animals in the mPFC/Cing (*n* = 7), Cing/RSP (n = 5), mPFC/RSP (*n* = 5) and RSP/RSP (*n* = 7) groups. The dashed horizontal lines signify the mean percentages while the vertical lines show the standard errors for each thalamic nucleus. See list of abbreviations.

Across the mPFC/Cing tracer pairings, the following percentages of double‐labelled cells represent the maximum found in the anteromedial nucleus (max ~6%), interanteromedial nucleus (max ~11%) and nucleus reuniens (max ~10%) (Figure 4). The anteromedial single‐labelled cells following tracer injections within the anterior cingulate cortex were often concentrated in the most medial and ventral parts of the nucleus, that is, close to interanteromedial nucleus. In the cases where double‐labelled cells were observed they were mostly scattered across the interanteromedial nucleus, as well as located in the medial and ventral parts of the anteromedial nucleus. The double‐labelled cells in nucleus reuniens were principally posterior to the anterior thalamic nuclei. In contrast, only one of the seven cases contained any single‐labelled neurons in either the anterodorsal or anteroventral nuclei following a tracer injection within the anterior cingulate cortex. Consequently, double‐labelled cells were not observed in either of these two nuclei.

When considered against the single‐labelled cells projecting to the medial prefrontal cortex, the highest proportion of bifurcating neurons was found within the interanteromedial nucleus. Likewise, the interanteromedial nucleus contained the highest proportion of double‐labelled cells when compared with those projecting to the anterior cingulate cortex (Table 1). For both measures, the anteromedial nucleus contained the lowest proportions of those nuclei with label from both injection sites (Table 1).

### 3.2 Collateral projections to both the anterior cingulate and retrosplenial cortices (Cing/RSP)

**FIGURE 5:**
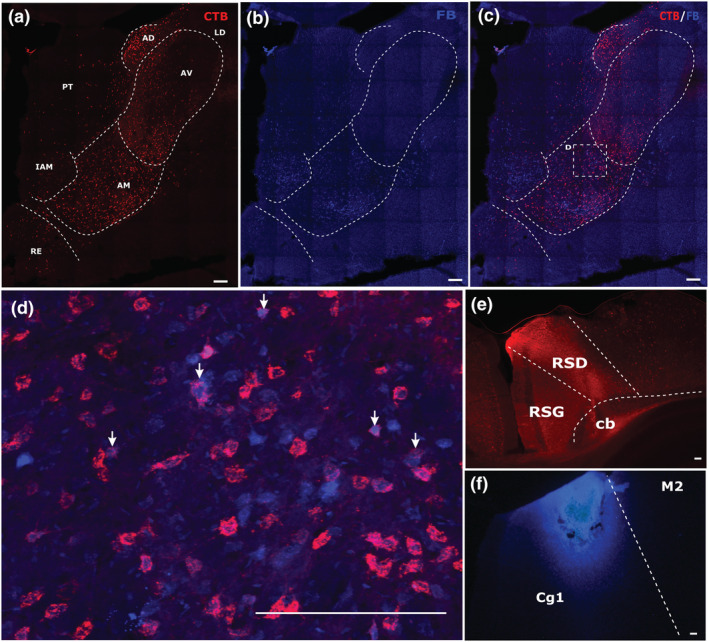
Example of combined retrosplenial (CTB) and anterior cingulate (FB) tracer injections (case 604#5). Coronal sections showing the target thalamic nuclei. Panels (a) and (b) show individual channel labels, and panel (c) shows the overlay of the two. Panels (e) and (f) show, respectively, the injections in the retrosplenial cortex and the anterior cingulate cortex. The enclosed area in panel (c) is magnified in panel (d). The arrows point to examples of double‐labelled cells, which show a blue centre (FB label) surrounded by a red halo (CTB label) within the AM. All scale bars are 150 μm. See list of abbreviations.

**FIGURE 6:**
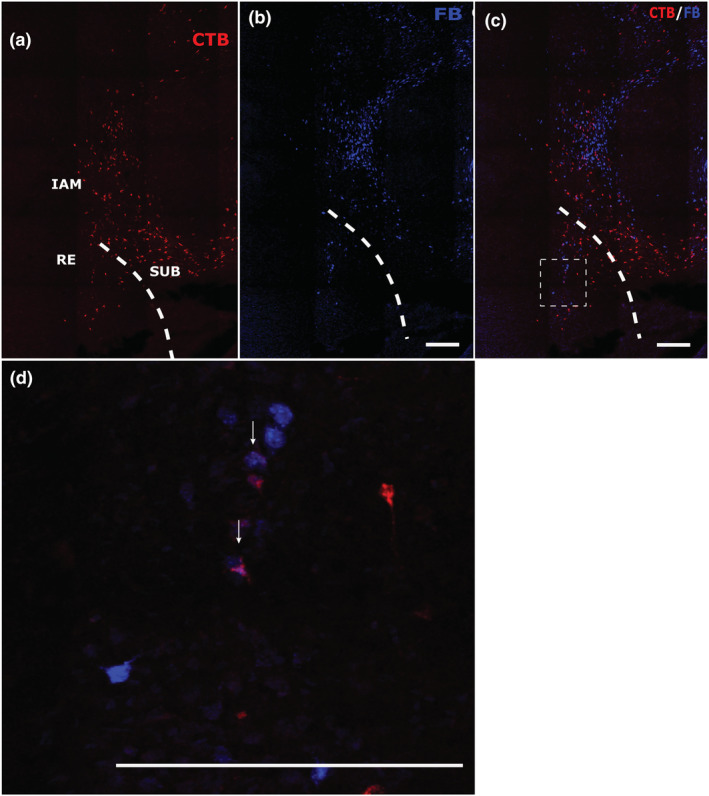
Labelling in nucleus reuniens in a retrosplenial/anterior cingulate pair (604#5). Panels in rows (a) and (b) show the individual channels with CTB and FB staining, and panel (c) shows the overlay of the two. The enclosed square in panel (c) is enlarged in panel (d). All scale bars are 150 μm. See list of abbreviations.

In five cases (Figures 1, 3 and 5), FB was injected into the anterior cingulate cortex and CTB in different portions of the retrosplenial cortex (AP: −3 to −5 mm). Like the mPFC/Cing pairs, albeit in slightly lower proportions, double‐labelled cells were observed within the anteromedial nucleus (max ~5%), the interanteromedial nucleus (max ~10%) and nucleus reuniens (max ~8%) (Figure 6). Again, double‐labelled cells were not observed in the anteroventral or anterodorsal nuclei, but very occasional double‐labelled cells were seen in the laterodorsal nucleus (max ~1.4%) (Figure 4).

The cell label following the injections in the anterior cingulate cortex was once again distributed mostly in medial and ventral portions of the anteromedial nucleus and in the interanteromedial nucleus. Following the tracer injections to retrosplenial cortex, single labelled cells were distributed across all three anterior thalamic nuclei. In cases with injections in the more anterior retrosplenial portions, there was very dense labelling within the anterodorsal nucleus. Single‐labelled cells were also scattered across the anteromedial and anteroventral nuclei. Within the anteroventral nucleus the single‐labelled cells were often densest close to the border with the anteromedial nucleus. Meanwhile, following the anterior cingulate injections there was a dense clustering of single‐labelled cells in the medial and most ventral portions of the anteromedial nucleus, where most of the double‐labelled cells within the nucleus were also found. Double‐labelled cells were also observed within nucleus reuniens, including cases where the tracers were positioned more rostrally in the anterior cingulate cortex and more caudally in the retrosplenial cortex, that is, were widely spaced. While label was often scattered across nucleus reuniens, the number of double‐labelled cells tended to increase in the more caudal parts of the nucleus, that is, posterior to the anterior thalamic nuclei.

Overall, relative to the number of cells projecting to the retrosplenial cortex, the proportion of bifurcating neurons was the highest for the interanteromedial nucleus and lowest for the anteroventral and anterodorsal nuclei, which appeared to contain no double‐labelled cells (Table 1). The laterodorsal nucleus had very low numbers of single‐labelled cells following the anterior cingulate injections, which were concentrated in the most ventral parts of the nucleus. In contrast, the retrosplenial injections resulted in single‐labelled cells in the dorsal parts of the laterodorsal nucleus. Consequently, there was very little overlap between the two areas of label, resulting in the very small proportion of double‐label around the mid‐depth of the nucleus. This very sparse double‐labelling in the laterodorsal nucleus was seen in those cases where a tracer was placed in the more caudal portions in the anterior cingulate cortex. Meanwhile, relative to the number of cells projecting to the anterior cingulate cortex, the anteromedial thalamic nucleus contained the highest proportion of double‐labelled cells (Table 1).

### 3.3 Collateral projections to both the medial prefrontal and the retrosplenial cortices (mPFC/RSP)

**FIGURE 7:**
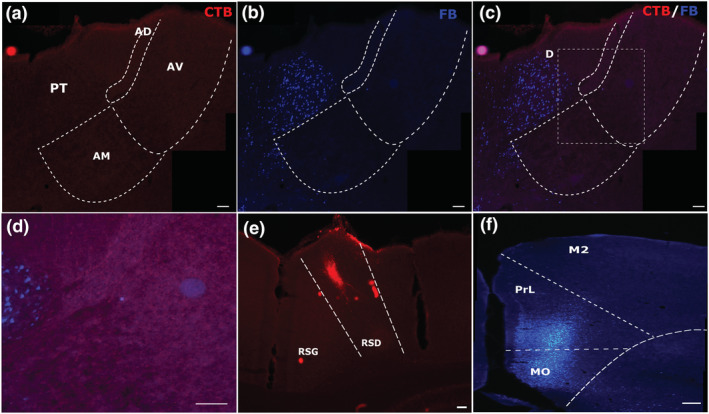
Example of combined medial prefrontal (mPFC) and retrosplenial cortex (RSP) injection pairing (case 223#3). Panels (a) and (b) show individual channels with CTB and FB cells following injections centred in the dysgranular retrosplenial cortex (e) and prelimbic/medial orbital cortices (f). Panel (c) shows the overlay of the two channels, and panel (d) shows the lack of double‐labelled cells. All scale bars are 150 μm. See list of abbreviations.

In five cases (Figures 1, 4 and 7) FB was injected into different portions of the medial prefrontal cortex (prelimbic, medial orbital and infralimbic) and CTB into different portions of the retrosplenial cortex (AP: −3.3 to −5.3 mm from bregma).

Following these pairs of injections, very low overall proportions of double‐labelled cells were seen in the anteroventral (max ~3%), anteromedial (max ~.13%) and interanteromedial (max ~.6%) nuclei, with no such cells being observed in some cases (Figure 7). The proportion of bifurcating neurons relative to single‐labelled cells was highest in the interanteromedial nucleus, with respect to cells projecting to the retrosplenial cortex, and the lowest within the anteromedial nucleus with respect to single‐labelled cells projecting to the mPFC (Table 1). Following tracer injections in the retrosplenial cortex there was a dense clustering of single‐labelled cells in the lateral portions of the anteroventral nucleus, with the rest of the label scattered across other portions of the anteroventral, anterodorsal and the anteromedial nuclei. At the same time, the single‐labelled cells following tracer injections in the mPFC were mostly in the anteromedial nucleus, interanteromedial nucleus and scattered within nucleus reuniens.

### 3.4 Collateral projections to different portions of retrosplenial cortex (RSP/RSP)

In seven cases the tracers FB and the CTB were infused in different portions of retrosplenial cortex. The injections were separated along the anterior–posterior axis (AP: −3 to −6.8 mm from bregma) with varying involvement of dysgranular and granular portions. In two cases (704#5 and 704#6) both injections were rostral to the splenium, that is, the rostral half of the overall area, but the tracer spread did not overlap (Figure 1).

**FIGURE 8:**
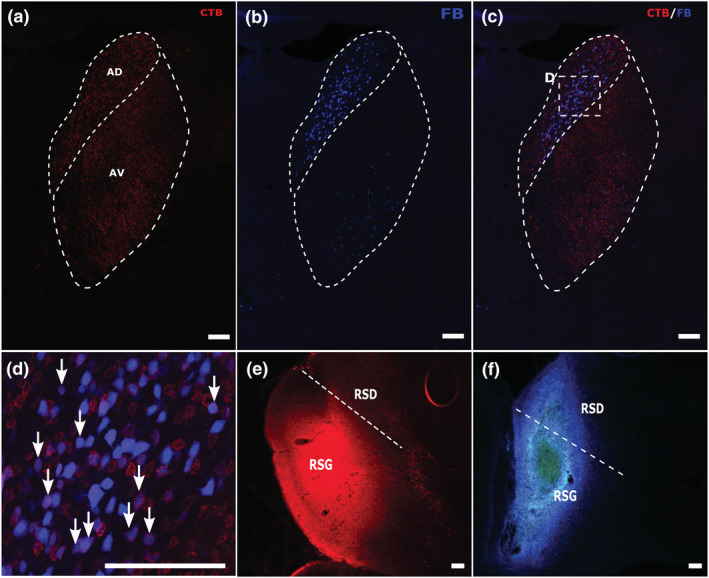
Example of combined retrosplenial cortex with retrosplenial cortex (RSP/RSP) injection pairing with double‐labelling in the anterodorsal nucleus (case 232#16). Panels A and B show individual channels for the CTB cells (a) that terminate in more caudal retrosplenial cortex and the FB cells (b) that terminate in the rostral retrosplenial cortex arising from within the anterodorsal and anteroventral nuclei. Panel (c) shows the overlay of both channels. Panel (e) shows the CTB injection site (caudal granular retrosplenial cortex). Panel (f) shows the FB injection site (rostral granular with some dysgranular retrosplenial cortex). Panel (d) is a magnified image of the boxed region in panel (c), showing the appearance of double‐labelled cells within the anterodorsal nucleus. Arrows point to some of the double‐labelled cells [blue centre (FB) surrounded by red halo (CTB)]. All scale bars are 150 μm. See list of abbreviations.

Unlike the previous injection pairings, the highest proportion of double‐labelled cells relative to the sum of all single‐labelled cells was in the anterodorsal nucleus (max ~15%) (Figures 1, 4, and 8). The proportion of double‐labelled cells within this nucleus appeared higher when the tracer was positioned in the more anterior portions of the retrosplenial cortex. The single‐labelling observed was often very dense and uniformly distributed across the anterodorsal nucleus regardless of the positioning of the tracers. Also, unlike the other injection pairings, there was double‐labelling in the anteroventral nucleus (max ~9%), although it was slightly less frequent than in the anterodorsal nucleus (Figure 4 and Table 1). Furthermore, the single‐labelled cells in the anteroventral nucleus showed a clear topography, as label was concentrated in a plexus along the ventrolateral border, in the rostral portions of the nucleus, but positioned closer to the anteromedial nucleus at more caudal levels. The single‐labelled cells from both the anterior and posterior retrosplenial injections were scattered and more uniformly distributed within the anteromedial nucleus, which may explain the lower proportion of double‐labelled cells (max ~6%; Figure 4), although this is comparable to the proportions in the Cing/mPFC and Cing/RSP pairings. Notably lower was the double‐labelling in the interanteromedial nucleus (max ~5%) and nucleus reuniens (max ~4%). Within nucleus reuniens, the single‐labelled cells following injections to both anterior and posterior retrosplenial cortex were scattered across all portions of reuniens, with very few double‐labelled cells (Table 1).

**FIGURE 9:**
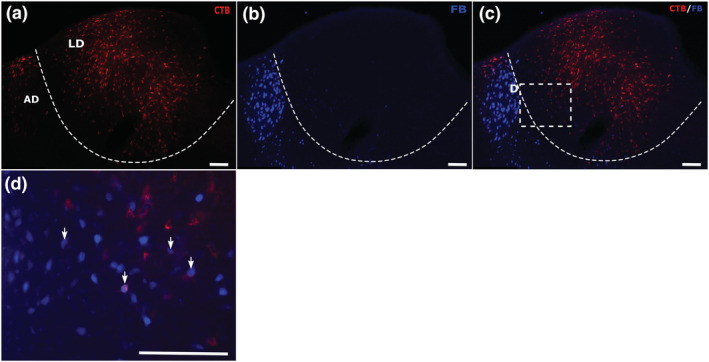
Example of laterodorsal nucleus labelling in a RSP/RSP pair (case 232#16). Panel (a) shows CTB labelling following an injection in the caudal retrosplenial cortex. Panel (b) left shows light FB labelling in the laterodorsal nucleus (alongside dense anterodorsal label) following an injection in the rostral retrosplenial cortex. Panel (c) shows the overlay of two. The enclosed square is magnified in panel (d) where arrows point the double‐labelled cells within the laterodorsal nucleus. All scale bars are 150 μm. See list of abbreviations.

Unlike the Cing/RSP pairing, there were double‐labelled cells in the laterodorsal nucleus. While the numbers of such cells remained low (max~3%; Figure 9), double‐labelled cells were seen in most cases. The projections from this nucleus were topographically organised as more posterior retrosplenial injections led to label in the most dorsal part of the laterodorsal nucleus while the more anterior retrosplenial injections led to label in its most ventral parts. This topography helped to separate the populations of labelled cells so that double‐labelled cells occurred at the point where these two single‐labelled populations of cells met.

### 3.5 Quantitative analyses

Friedman tests were conducted within each pair of injections to compare the distributions of the proportion of double‐labelled cells between the nuclei of interest. The overall Friedman tests revealed statistically significant differences between the distributions of double‐labelled cells in two of the groups: (1) mPFC/Cing: *χ* ^2^(4) = 14.41, *p* = .006. Initial *post hoc* comparisons revealed statistically significant differences between the anterodorsal and interanteromedial nuclei (*p* = .011) and the anteroventral and interanteromedial nuclei (*p* = .011) double‐labelled proportion distributions, however these comparisons did not survive the Bonferonni multiple comparison adjustments (*p*_*s*_ = .11); (2) RSP/RSP: *χ* ^2^(5) = 18.68, *p* = .002. Initial *post hoc* analyses revealed statistically significant differences between the interanteromedial and anteroventral nuclei (*p* = .018), interanteromedial and anterodorsal nuclei (*p* < .001), nucleus reuniens and anteroventral nucleus (*p* = .022), nucleus reuniens and the anterodorsal nucleus (*p* < .001), laterodorsal nucleus and anterodorsal nucleus (*p* = .022) and the anteromedial and anterodorsal nuclei (*p* = .038). Of these, the differences in the distributions between the interanteromedial and anterodorsal nuclei (*p* = .007) and the nucleus reuniens and anterodorsal nucleus (*p* = .009) survived the Bonferroni multiple comparison correction. There was no overall model effect in the mPFC/RSP pair, *χ* ^2^(4) = 2, *p* = .74 or in the Cing/RSP pair, *χ* ^2^(5) = 8.5, *p* = .13.

### 3.6 Further appraisal of double‐labelled cell counts

Tissue from three selected cases, each with relatively high counts of double‐labelled cells were investigated using confocal microscopy. These additional analyses informed several issues. Despite the thickness of the sections (50 μm), there was no evidence of false‐positive counting in the main study as, overall, the labelled cell counts from the confocal images were often higher. Nevertheless, the rank order of proportions of double‐labelled cells across different thalamic nuclei remained little changed. In one case (225#8; mPFC/Cing) the fluorescent data and confocal data were closely matched across all nuclei. In a second case (232#16; RSP/RSP) there were very small increases in the proportions of double‐labelled cells, with the exception of AD (rising from 13.7% to 21.8%). However, the Cing/RSP case (604#5) gave higher proportions of double‐labelled cells for almost all nuclei (most notably for IAM rising to 21.3% and AM to 11.4%). These additional analyses indicate that the fluorescent cell counts reflected the rank order of collateral projections across nuclei, but the method can underestimate labelled‐cell numbers, including the upper limit of double‐labelled cells.

## 4 DISCUSSION

To help understand how rostral thalamic nuclei influence medial cortical areas, pairs of tracers were injected into specific areas within the medial prefrontal and retrosplenial cortices. Retrograde label was then examined in the three principal anterior thalamic nuclei, the interanteromedial nucleus, nucleus reuniens and the laterodorsal nucleus. In addition to confirming previously established thalamo‐cortical projections (Condé et al., 1990; Horikawa et al., 1988; Shibata, 1993; Vertes, 2004), two patterns of bifurcating projections were observed. Neurons innervating both medial prefrontal/anterior cingulate cortices and anterior cingulate/retrosplenial cortices were most evident at or close to the thalamic midline. In contrast, neurons that collaterise to reach different parts of retrosplenial cortex were most frequent in the anterodorsal thalamic nucleus, although all selected thalamic nuclei contained some bifurcating neurons that simultaneously reach different parts of retrosplenial cortex. A third category was represented by a lack of collateralisation, for example, very few thalamic neurons were observed that project to both medial prefrontal and retrosplenial cortices.

One task was to resolve whether neurons from the anterior thalamic nuclei collaterise to reach different parts of retrosplenial cortex. Our results closely follow one study (Horikawa et al., 1988) by finding a modest minority of such anterior thalamic cells, but contrast with another (Sripanidkulchai & Wyss, 1986) that reported no such collaterals. In view of these differences, we should consider the possibility of false positives in our study (and that of Horikawa et al., 1988). Potential causes would include an overlap between pairs of adjacent cortical injections or direct tracer uptake by the cingulum bundle (Bubb et al., 2020; Domesick, 1970). The former explanation (direct spread) appears unlikely given the wide spacing of the retrosplenial injections in this and the previous study (Horikawa et al., 1988). To test for the second explanation (direct cingulum body uptake) we examined both the injection sites and the topography of retrogradely labelled cells within the anterior thalamic nuclei to see if they matched previous descriptions for that part of the retrosplenial cortex (Bubb et al., 2020; Lomi et al., 2021; Perry et al., 2021; Shibata, 1993; Sripanidkulchai & Wyss, 1986) or whether the labelled cells were distributed broadly across the nuclei, that is, more consistent with cingulum uptake. By these measures we did not find evidence of direct cingulum bundle uptake. Consequently, we conclude that there is a modest population of anterior thalamic neurons with collaterals that reach widely separated parts of retrosplenial cortex (see also Horikawa et al., 1988). This conclusion can be extended as an earlier report noted that ~10% of labelled anteromedial nucleus neurons project to both the rostral and caudal portions of anterior cingulate cortex (Horikawa et al., 1988).

Of the anterior thalamic nuclei, the anterodorsal nucleus typically contained the highest proportion of retrosplenial/retrosplenial collaterals, often in modest numbers, although confocal microscopy indicated that this proportion might reach 22%. A very similar preponderance in the anterodorsal nucleus (reaching 21%) was previously reported (Horikawa et al., 1988). Together, these findings (Figure 4) reinforce other differences between the three major anterior thalamic nuclei (Aggleton et al., 2010; Byatt & Dalrymple‐Alford, 1996; Phillips et al., 2019; Safari et al., 2020). The anterodorsal nucleus is a key element of the ‘head‐direction system’ (Taube, 1995), providing compass‐like signals and assisting navigation (Taube, 2007). This nucleus is heavily interconnected across the retrosplenial cortex, a cortical region also importantly involved in spatial memory and navigation (Cooper & Mizumori, 2001; Harker & Whishaw, 2004; Mitchell et al., 2018; Nelson et al., 2015; Vann et al., 2009; Wolbers & Büchel, 2005). One implication is that the information provided by the rat's current heading direction can simultaneously influence diverse areas of retrosplenial cortex, reflecting the relevance of this information for on‐line navigation.

In contrast, very few bifurcating neurons originated in the laterodorsal nucleus to reach different parts of retrosplenial cortex, consistent with a previous study (Horikawa et al., 1988). This contrast with the anterodorsal nucleus is all the more striking as both thalamic nuclei contain numerous head‐direction cells (Mizumori & Williams, 1993; Taube, 2007) and both project to the granular and dysgranular retrosplenial cortices (Shibata, 1993; Sripanidkulchai & Wyss, 1986; van Groen & Wyss, 1990; Wyss & Van Groen, 1992). But, unlike the anterodorsal nucleus, the laterodorsal nucleus does not receive direct head‐direction information from the lateral mammillary bodies (Dillingham et al., 2015). Rather, the laterodorsal nucleus receives a greater array of cortical and subcortical visual inputs than the anterior thalamic nuclei (Bezdudnaya & Keller, 2008). Consequently, it has been argued that the laterodorsal nucleus head‐direction neurons have qualitatively different properties from those in the anterodorsal nucleus (Dudchenko et al., 2019). One of these different properties appears to be the nature of their inputs to retrosplenial cortex.

Our findings also align with previous descriptions of collateral projections from the anterior and midline thalamic nuclei that reach both prefrontal and anterior cingulate areas (Condé et al., 1990) as well as those that reach both anterior cingulate and retrosplenial areas (Horikawa et al., 1988). Like the former study (Condé et al., 1990), we observed double‐labelled cells in the mPFC/Cing cases within those thalamic nuclei at or adjacent to the midline, that is, nucleus reuniens and the interoanteromedial nucleus. The proportion of double‐labelled cells previously reported (Condé et al., 1990) in nucleus reuniens (~8% of the less frequent efferent) appears comparable to that in the present study (Table 1). Nevertheless, the absolute numbers of labelled cells in nucleus reuniens following medial prefrontal injections appeared lower than in some previous studies (e.g., Hoover & Vertes, 2007; Vertes et al., 2006). A partial explanation is that the counting method tended to provide conservative counts, as indicated by the confocal data. The present study extended that of Condé et al. (1990) by describing double‐labelled cells in the anteromedial nucleus and including a wider combination of prefrontal areas to receive tracer injections. Meanwhile, Condé et al. (1990) also reported double‐labelled cells in the ventromedial thalamic nucleus, rhomboid nucleus and mediodorsal thalamic nucleus. Remarkably, the many double‐labelled cells in select lateral part of the mediodorsal nucleus reached up to 90% of the neurons labelled by one of the tracers (Condé et al., 1990). This striking difference between the properties of the anterior thalamic nuclei (limited bifurcation) and mediodorsal nucleus (considerable bifurcation) highlights how these integral parts of the ‘cognitive thalamus’ have contrasting roles (Aggleton et al., 2010; Clark & Harvey, 2016; Pergola et al., 2018; Perry et al., 2021; Sweeney‐Reed et al., 2021). While the parallel mediodorsal nucleus efferents are more consistent with a regulatory role across multiple prefrontal functions (Mitchell & Chakraborty, 2013; Pergola et al., 2018), those from the anterior thalamic nuclei imply the conveyance of more specific information, for example, relating to space (O'Mara & Aggleton, 2019).

The proportions of anterior thalamic neurons reaching both the anterior cingulate and retrosplenial cortices in the present study appeared slightly lower than those in a previous study (Horikawa et al., 1988). That study reported how, within the anterior thalamic nuclei, the anteromedial nucleus contained the highest proportion (~13%) of double‐labelled cells projecting to both anterior cingulate and retrosplenial cortices (Horikawa et al., 1988). In the present study, the anteromedial nucleus again contained the highest proportion from the three principal anterior thalamic nuclei (~6% but reaching 11% in the confocal case). Meanwhile, the adjacent interanteromedial nucleus and nucleus reuniens contained higher proportions (~9%). The double‐label in the interanteromedial nucleus is informative as Horikawa et al. (1988) did not separate this area from the anteromedial nucleus, partly explaining their higher counts. In both studies, the anterodorsal and anteroventral nuclei contained no labelled neurons reaching both the rostral anterior cingulate and retrosplenial cortex, while the laterodorsal nucleus contained <2% of labelled cells. It was the case, however, that injections involving the caudal anterior cingulate cortex and retrosplenial cortex (Horikawa et al., 1988) led to modest numbers of double‐labelled cells also being observed in the other anterior thalamic nuclei. This apparent difference from the present study may reflect how those more caudal anterior cingulate injections involved the midcingulate area 24′ (Vogt & Paxinos, 2012). This transition area, which had not been distinguished at the time of the earlier study, was deliberately avoided in the present study.

A largely complementary study also investigated collateral cortical projections from the anteromedial nucleus and nucleus reuniens (Pei et al., 2021). Much of their focus was on whether cortical collaterals reach the hippocampal formation (Pei et al., 2021). Consequently, the study included medial prefrontal cortex/dorsal subiculum, medial prefrontal cortex/ventral subiculum, caudal retrosplenial/dorsal subiculum and caudal retrosplenial/ventral subiculum injection pairings. All injection pairings led to double‐labelled cells in the two thalamic nuclei, with the highest proportions in the anteromedial nucleus and nucleus reuniens for the medial prefrontal/dorsal subiculum pairing (both >30% of all label). Finding an appreciable proportion of neurons in nucleus reuniens that project to both medial prefrontal cortex and the hippocampal formation (~36%) appears inconsistent with the 5%–10% previously reported for the same termination pairings (Hoover & Vertes, 2012; Varela et al., 2014). Furthermore, it had been observed that considerably more double‐labelled cells are present in the ventral hippocampal formation (Hoover & Vertes, 2012), something not described in the later study (Pei et al., 2021). In addition, Pei et al. (2021) reported appreciable populations of double‐labelled cells in both the anteromedial nucleus (~20%) and nucleus reuniens (26%) for the medial prefrontal cortex/retrosplenial cortex pairing (Pei et al., 2021), yet this same combination gave proportions closer to zero in the present study.

Overall, the study by Pei et al. (2021) produced higher proportions of double‐labelled thalamic cells than reported in previous analyses of similar target pairings (Condé et al., 1990; Horikawa et al., 1988; Varela et al., 2014), including the present one. One potential explanation for these higher proportional cell counts is that Pei et al. (2021) focused on those cases with more extensive tracer injections within the target areas. (Until both target areas are filled with tracer, the resulting double‐cell counts will always be an underestimate.) One unintended consequence, however, was that ‘medial prefrontal’ cortex injections extended into anterior cingulate cortex (Pei et al., 2021), a factor that might increase the proportions of double‐labelled cells within the anteromedial nucleus and nucleus reuniens (see anterior cingulate/retrosplenial pairs here and Horikawa et al., 1988). Furthermore, their cell counts (Pei et al., 2021) just involved a restricted subarea of each target nucleus. This method will give higher double‐cell counts if there is a bias towards zones of label overlap. In contrast, the present study counted cells across each entire nucleus, a method likely to reduce the proportions of double‐labelled cells given the topographic origins of many of the cortical projections from within the target nuclei (Lomi et al., 2021; Shibata, 1993; Sripanidkulchai & Wyss, 1986). Even though the counting methods in the present study tended to be conservative (as indicated by parallel confocal analyses), when cell counts are made across the entire nucleus (Condé et al., 1990; Horikawa et al., 1988; Varela et al., 2014) the proportions of double‐labelled cells were more comparable to those in the present study.

Consistent with previous studies, the distribution of single‐labelled cells highlights how rostral thalamic nuclei and those close to the midline project to a wide array of frontal and cingulate sites, a pattern seen not only in rats (Condé et al., 1990; Van Der Werf et al., 2002; Vertes, 2004), but also in non‐human primates (Barbas et al., 1991; Kievit & Kuypers, 1977). One goal of the present study was to compare the properties of efferents from nucleus reuniens with those from the anterior thalamic nuclei. Both sites are interconnected with many of the same sites and both are presumed to make important contributions to cognition (Aggleton et al., 2010; Cassel et al., 2021; Griffin, 2021; Mathiasen et al., 2021, 2020; Pei et al., 2021). Both the anteromedial nucleus and nucleus reuniens contain a similar modest proportion of thalamo‐cortical neurons that innervate multiple frontal areas (see also Condé et al., 1990). A small minority of bifurcating projections is also found when looking for individual prefrontal neurons that reach both nucleus reuniens and the anterior thalamic nuclei (Mathiasen et al., 2021), though some prefrontal inputs to nucleus reuniens instead collaterise to reach medial temporal sites (Schlecht et al., 2022; Varela et al., 2014). The dominant pattern of input separation is highlighted by the anterior thalamic nuclei as very few neurons simultaneously project to both the anteromedial and anteroventral nuclei (Wright et al., 2013). A further thalamic site deserving additional interest is the parataenial nucleus. Like some of the other nuclei under investigation, the parataenial nucleus gives rise to extensive prelimbic and infralimbic projections (Figures 2, 5 and 7; see also Van Der Werf et al., 2002; Vertes & Hoover, 2008). The same region has also been highlighted in some post‐mortem studies of Korsakoff's syndrome (Mair et al., 1979; Mayes et al., 1988).

The observed bias to neuronal separation is consistent with the prevailing view that the various anterior thalamic nuclei and nucleus reuniens operate in parallel, complementary ways that reflect subtle topographic and functional differences (Aggleton et al., 2010; Ferraris et al., 2021; Griffin, 2021; Mathiasen et al., 2021, 2020). These same results also highlight the presence of parallel prefrontal—thalamic—hippocampal pathways, one involving nucleus reuniens, the other the anterior thalamic nuclei (Prasad & Chudasama, 2013). Within the anterior thalamic nuclei, the relative rates of collaterisation strengthen proposals concerning the respective functions of its principal nuclei (Aggleton et al., 2010), with the anteromedial (and interanteromedial) nucleus reflecting prefrontal attributes, while the anteroventral and anterodorsal nuclei are more closely linked with retrosplenial and hippocampal formation functions (Aggleton & O'Mara, 2022; Yamawaki et al., 2019).

## CONFLICT OF INTEREST

The authors declare that there is no conflict of interest.

## AUTHOR CONTRIBUTIONS

S. Y.: tracer injections, histological analysis, data analysis and manuscript preparation; M. L. M.: tracer injections and manuscript preparation; E. A.: histological analyses; A. J. D. N.: additional tracer injections; S. M. O'M.: funding and manuscript preparation; J. P. A.: funding, experimental design and manuscript preparation.

### PEER REVIEW

The peer review history for this article is available at https://publons.com/publon/10.1111/ejn.15819.

## Data Availability

Available on request to the communicating author.
